# Night-Time Noise Index Based on the Integration of Awakening Potential

**DOI:** 10.3390/ijerph13030272

**Published:** 2016-03-01

**Authors:** Junta Tagusari, Tomoya Takashima, Satoshi Furukawa, Toshihito Matsui

**Affiliations:** 1 Graduate School of Engineering, Kyoto University, Kyoto 615-8530, Japan; junta.tagusari@gmail.com (J.T.); firstchildren_sessions@yahoo.co.jp (T.T.); furukaws@pref.okinawa.lg.jp (S.F.); 2 Graduate School of Engineering, Hokkaido University, Sapporo 060-8628, Japan

**Keywords:** night-time noise, sleep, sleep disturbance, awakening, neurophysiology, Phillips-Robinson model

## Abstract

Sleep disturbance induced by night-time noise is a serious environmental problem that can cause adverse health effects, such as hypertension and ischemic heart disease. Night-time noise indices are used to facilitate the enforcement of permitted noise levels during night-time. However, existing night-time noise indices, such as sound exposure level (SEL), maximum sound level ($L_{\mathrm{Amax}}$) and night equivalent level ($L_{\mathrm{night}}$) are selected mainly because of practical reasons. Therefore, this study proposes a noise index based on neurophysiological determinants of the awakening process. These determinants have revealed that the potential on awakening is likely integrated into the brainstem that dominates wakefulness and sleep. From this evidence, a night-time noise index, $N_{\mathrm{awake},\mathrm{year}}$, was redefined based on the integration of the awakening potential unit ($p_{\mathrm{unit}}$) estimated from the existing dose-response relationships of awakening. The newly-defined index considers the total number of awakenings and covers a wide-range and number of noise events. We also presented examples of its applicability to traffic noise. Although further studies are needed, it may reveal a reasonable dose-response relationship between sleep disturbance and adverse health effects and provide a consistent explanation for the risks of different sound sources where the characteristics of noise exposure are quite different.

## 1. Introduction

### 1.1. Noise-Induced Sleep Disturbance and Indices of Night-Time Noise

Sleep disturbance induced by night-time noise is a serious environmental problem with associated health concerns. The World Health Organization (WHO) Regional Office for Europe [1] estimated the disability-adjusted life years (DALYs) lost from sleep disturbance to be 903,000 years for the 285 million population living in agglomerations with >50,000 inhabitants. This DALYs value is considered to be relatively high, while the total DALYs lost in Europe was estimated to be 151,461,000 years for the 883 million population there [2].

While night-time noise was evaluated based on “self-reported sleep disturbance” in this study, night-time noise may also cause objective sleep disturbance where awakenings in response to specific noise events occur repeatedly. Both subjective (self-reported) and objective (evaluated by awakenings) sleep disturbances are environmental problems that can cause adverse health effects, such as hypertension and ischemic heart disease [3,4,5,6]. The WHO Regional Office for Europe considered their health implications and developed a guideline for night-time noise [4].

Night-time noise-induced awakening (objective sleep disturbance) is measured and defined in numerous ways, such as polysomnography, an actimeter and a button that provides a prescribed signal of awakening [7].

Polysomnography remains the gold standard for evaluating sleep structures. Awakening is measured using polysomnography and defined as arousal (short (≥3 s) and unconscious electric activation) and electroencephalogram (EEG) awakening (sleep stage change to the wake states) [7]. Few field studies have been carried out using polysomnography, because it is expensive and may influence sleep. However Basner *et al.* [8] performed a field study using polysomnography, which showed a dose-response relationship between sound levels and the probability of EEG awakening.

In several field studies, awakenings have been measured using an actimeter (motility and onset of motility) and by pressing a button (behavioral awakening). Motility and onset of motility are non-invasive measurements of body movement performed during sleep using an actimeter [9,10], and they are closely related to sleep duration and onset, as well as waking time [11]. In addition, the number of body movements and EEG awakenings are correlated [8]. However, awakenings can occur without body movements, while there are also body movements without awakening, which limit the validity of the measurements [7].

Behavioral awakening is measured by the pressing of a button (or other ways that provide a prescribed signal) by a subject awakened from sleep, which directly relates to conscious awakenings, and has been used in numerous field studies [12,13,14,15]. However, the number of behavioral awakenings would be much smaller than the EEG awakenings measured using polysomnography, because consciousness is only regained following prolonged wake periods, which reduces its reliability [7]. Passchier-Vermeer *et al.* [15] reported a dose-response relationship between sound levels of aircraft noise and the probability of behavioral awakening, which shows that the probability of awakening is very low even when the sound level is high. Although this measurement has a disadvantage, behavioral awakening is considered to be an important measurement related to sleep disturbance, because it has been used in several field studies, directly relates to awakening during sleep and is the strongest form of sleep activation.

Night-time noise indices are used to facilitate the enforcement of permitted noise levels during sleep based on subjective and objective sleep disturbances. Noise indices such as sound exposure level (SEL), maximum sound level ($L_{\mathrm{Amax}}$) and night equivalent level ($L_{\mathrm{night}}$) are widely used empirically to evaluate a single noise event or overall noise exposure during night-time.

Both the SEL and $L_{\mathrm{Amax}}$ have been used for evaluating and predicting the probability of awakening in response to a single noise event. The SEL has been widely used in field studies [9,12,13,14] because it provides information on the duration of a noise event and shows a higher correlation with awakening than the $L_{\mathrm{Amax}}$ [16]. However, no reasonable explanations could be provided for the sum of the sound power being over the noise event. Meanwhile, the $L_{\mathrm{Amax}}$ that is reported to be highly correlated with sleep stage changes [16] has also been used in several studies [8]. These indices have been introduced and used for evaluating and predicting an awakening in response to a single noise; however, noise-induced sleep disturbances and adverse health effects are most likely to be caused by long-term noise exposure.

The $L_{\mathrm{night}}$ has been used to evaluate and predict the long-term effects of night-time noise exposure [4,17], which is reported to be associated with self-reported sleep disturbance [1,10]. However, it was introduced for empirical reasons. Since the $L_{\mathrm{night}}$ sums up the sound power during night-time, the $L_{\mathrm{night}}$ gains only 3 dB when the number of noise events doubles. The number of awakenings in response to noise events is remarkably different depending on the number of noise events even if the $L_{\mathrm{night}}$ is the same value, which suggests that the number of noise events should be accounted for [4,7]. To solve this problem, the total number of awakenings ($N_{\mathrm{awake},\mathrm{year}}$) index was proposed based on the probability of awakening in response to a single noise event [18,19]. Moreover, Janssen *et al.* [10] included the SEL and the logarithm of the number of noise events simultaneously into the statistical analysis of the number of noise events in sleep disturbance measured using motility and concluded that the number of noise events is largely represented in the $L_{\mathrm{night}}$. However, no effect of the number of events was found after the sleep period was accounted for in the statistical analysis.

In addition to noise indices and the number of noise events, the distribution and sound source of noise events are considered to affect sleep disturbance [7,20]. To account for the distribution of noise events during sleep, time indicators such as time after falling asleep and sleep period, have been included in the statistical analysis in numerous studies [8,10,18,21]. The American National Standards Institute (ANSI) [18] defined a dose-response relationship between the probability of behavioral awakening due to aircraft noise and SEL levels, where the time after falling asleep was considered, which estimates the probability of awakening as much higher than the value based on Passchier-Vermeer’s dose-response relationship [15]. The effect of sound sources on sleep is less clear, although sleep disturbance due to aircraft noise has been reported to be higher than road traffic noise and railway noise under the same noise level conditions [4,20].

We should note that since night-time noise studies are focused on noise-induced sleep disturbance and sleep is a physiological function of the human body and a neurophysiological function of the brain, these noise indices and confounding factors should be validated neurophysiologically. However, especially regarding noise indices, the existing indices are not based on neurophysiological findings of the awakening process and are mainly applied for practical reasons, which means they might be inappropriate for evaluating night-time noise. We [22] investigated the dynamic characteristics of the brainstem dominating sleep and wakefulness, which suggested that the validity of the $L_{\mathrm{Amax}}$ and SEL were limited as short-term noise indices for different durations of noise events. A neurophysiologically-validated index for night-time noise should be proposed to evaluate and predict noise-induced sleep disturbance.

### 1.2. Neurophysiology of Sleep and Awakening

Neurophysiologically, the modulation of wakefulness and sleep is dominated by the ascending arousal system (AAS) nuclei in the brainstem and the ventrolateral preoptic (VLPO) nuclei in the hypothalamus [23]. Furthermore, circadian and homeostatic drives control the activities of the nuclei, and the sleep-awake switch is characterized by mutual inhibition by the nuclei [23].

Numerous mathematical models have been developed for explaining the activation of the nuclei in the brainstem, such as the two-process model [24] and the mutual inhibition model [25]. The two-process model explains the sleep-wake cycle based on the two processes, which are the homeostatic and circadian processes that increase sleep pressure and modulate the threshold of falling asleep during wakefulness, respectively. In addition, a mutual inhibition model was developed based on the subsequent physiological findings of the interaction in the brainstem, which revealed that the monoaminergic (MA) nuclei in the AAS was activated during wakefulness and the VLPO nuclei during sleep, while both nuclei mutually inhibit the activation of the other. These models not only showed a calculated example that agreed with existing evidence, but also provided a helpful explanation of the dynamics of sleep and wake, which shows significant insights into awakening due to external stimuli [26,27], effects of caffeine [28] and chronotype [29].

**Figure 1:**
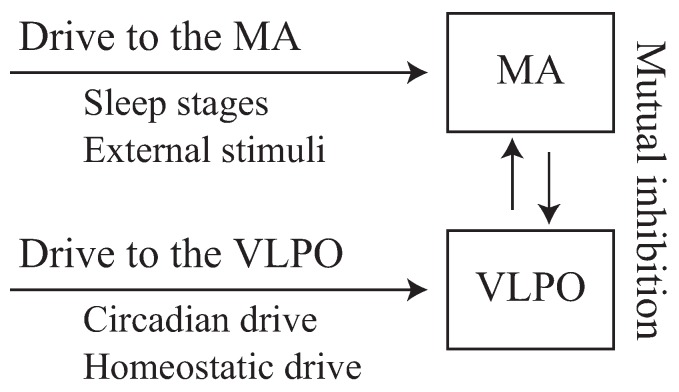
Illustration of schematic diagram of the Phillips-Robinson model. Monoaminergic (MA) and ventrolateral preoptic (VLPO) nuclei are activated by the external drives related to sleep stages and external stimuli, as well as circadian and homeostatic drives. MA and VLPO nuclei mutually inhibit each other, which constitutes the sleep-awake switch.

The Phillips-Robinson model [25] is a mutual inhibition model, which explains the neurophysiological dynamics of the brainstem. Only two numerical populations were included in this simple mathematical model, namely the MA nuclei in the AAS, which is activated during wakefulness and inactivated during sleep, and the VLPO nuclei, which exhibits the opposite effects. The schematic diagram of this model is shown in Figure 1. This model enables the quantitative evaluation of the sleep-awake switch, as well as any brief awakening due to external stimuli [26,27]. However, the ultradian rhythm was neglected, and rapid eye movement (REM) sleep is not explained in this model, which is also used for evaluating the various effects on sleep, suggesting that this model is useful for understanding physiological response during sleep and the structure of the sleep.

Based on the Phillips–Robinson model, awakening of the brainstem could be defined, which lasts at least tens of seconds [26,27]. Awakening of the brainstem is assumed to relate to measurable awakenings, such as motility, EEG and behavioral awakening, which may affect the endocrine system and cause adverse health effects. We investigated the relationship between the neuro-electrical thresholds of the awakenings and the duration of the external stimuli input into the brainstem [22]. In addition, the thresholds were converted to sound levels based on a previously-reported laboratory experiment [30].

The calculated threshold levels of awakening gave the following results:

1. The brainstem integrates awakening potential, but not the sound energy of the external stimuli.
2. The brainstem integrates the potential with a first-order lag system and a time constant of approximately 10–100 s.
3. The threshold levels of awakening due to short-duration noises are extremely high, while the $L_{\mathrm{Amax}}$ and SEL both overestimated this parameter.
4. The SEL index overestimates even for long-duration noises because the brainstem integrates the awakening potential with a time constant of 10–100 s.

These results suggest that the existing night-time noise indices, SEL, $L_{\mathrm{Amax}}$ and $L_{\mathrm{night}}$, are not appropriate for the evaluation and prediction of awakening response, particularly because they tend to overestimate evaluations of the awakening response.

Night-time noise indices and confounding factors were introduced empirically, although they were intended to evaluate the effect of noise on sleep that is a neurophysiological function of the brain. There are numerous neurophysiological findings on sleep and wakefulness, and moreover, the implementation of the mathematical Phillips–Robinson model has revealed the dynamics of the brainstem that integrates awakening potential, but not the sound energy. Therefore, in this study, we introduced the awakening potential unit ($p_{\mathrm{unit}}$) based on the results mentioned above and those of existing studies on the relationship between noise and the probability of awakening. Furthermore, we define a night-time noise index, $N_{\mathrm{awake},\mathrm{year}}$, using the awakening potential, which can be obtained from night-time sound level fluctuations and shows the expected number of awakenings (or probability of awakening) per year. This index is based on neurophysiological findings of sleep and covers a wide-range of night-time noise events. We also presented examples of the applicability of the $N_{\mathrm{awake},\mathrm{year}}$ index to traffic noise.

## 2. Method

### 2.1. Introduction of $p_{\mathrm{unit}}$

In this section, we redefined the night-time noise index, $N_{\mathrm{awake},\mathrm{year}}$, by introducing the $p_{\mathrm{unit}}$, which is based on the results of the simulated calculation of the Phillips–Robinson model mentioned in the previous section.

The external neuro-electrical stimuli to the brainstem, D(t) (mV), is assumed to be a function, *f*, of the sound stimuli as follows:

(1) D(t)=f(L(t))

where L(t) (dB) is the indoor sound level fluctuation of a single noise event, with *t* as the time in seconds.

The external stimuli, D(t), is approximately integrated with a first-order lag system in the brainstem. The integrated potential at $t_{0}$, $\bar{D}\left(t_{0}\right)$ is expressed by the following equation:

(2) $$\bar{D}\left(t_{0}\right)=\int_{-\infty }^{t_{0}}e^{-(t_{0}-t)/\tau }D\left(t\right)\;dt$$

where *τ* is the time constant (10–100 s) of the lag system.

Most traffic noise that occurs during the night-time is considered a single event because there is less traffic volume then. If the duration of the noise event is relatively shorter than the time constant of the brainstem, *τ*, then the maximum value of $\bar{D}\left(t\right)$ due to the single noise event can be approximated using the following equation:

(3) $${\bar{D}}_{\max}∼\int_{-\infty }^{\infty }D\left(t\right)\;dt$$

The awakening risk would correlate with the value of ${\bar{D}}_{\max}$, and therefore, the dose-response relationship with awakening should be expressed as a function of ${\bar{D}}_{\max}$. Following the introducing of a function, *g*, which depicts the dose-response relationship between the probability of awakening and ${\bar{D}}_{\max}$, the probability of awakening due to a single noise event ($P_{\mathrm{single}}$) may be formulated as:

(4) $$\begin{array}{cc} P_{\mathrm{single}} & =g\left({\bar{D}}_{\max}\right) \end{array}$$

(5) $$\begin{array}{cc} & =g\left(\int_{-\infty }^{\infty }D\left(t\right)\;dt\right) \end{array}$$

Under the assumption that the function *g* has linearity (additivity), the expression of $P_{\mathrm{single}}$ can be transformed into:

(6) $$\begin{array}{cc} P_{\mathrm{single}} & =\int_{-\infty }^{\infty }g\left(D(t)\right)\;dt\\ & =\int_{-\infty }^{\infty }g\left(f(L(t))\right)\;dt \end{array}$$

This equation is further simplified by substituting $p_{\mathrm{unit}}\left(L\left(t\right)\right)$ for g(f(L(t))),

(7) $$P_{\mathrm{single}}=\int_{-\infty }^{\infty }p_{\mathrm{unit}}\left(L\left(t\right)\right)\;dt$$

where the function, $p_{\mathrm{unit}}\left(L\left(t\right)\right)$ (s${}^{-1}$), is interpreted as a unit potential of awakening per second at L(t) (dB).

Equation (7) means that the probability of awakening is calculated by the integral of the awakening potential. This is fundamentally different from the existing indices where the probability of awakening is calculated using the $L_{\mathrm{Amax}}$ or the SEL.

The function, $p_{\mathrm{unit}}\left(L\left(t\right)\right)$, should be determined using a field study or a laboratory experiment, and in this study, three existing dose-response relationships involving awakening were used in the estimation:

1. Passchier-Vermeer [15] reported a dose-response relationship between the probability of behavioral awakening and the SEL of a single noise event, expressed as Equation (8). This relationship is considered to be important, because it was based on field studies including a recent field study [9,10], where motility and self-reported sleep disturbance were also measured, and the number of noise events was considered to be represented in the $L_{\mathrm{night}}$ when motility is used for evaluating sleep disturbance. In addition, this Passchier-Vermeer relationship was used to establish the “Night Noise Guideline for Europe” by the WHO Regional Office for Europe [4].
2. The ANSI [18] defined a dose-response relationship between the probability of behavioral awakening and SEL, expressed as Equation (9). This relationship, which was authorized for use by the ANSI, considered the effect of the elapsed time after falling asleep, and therefore, the probability of awakening was calculated to be higher than that calculated using Passchier-Vermeer’s equation.
3. Basner [8] reported a dose-response relationship between the probability of EEG awakening and $L_{\mathrm{Amax}}$, expressed as Equation (10). This relationship was based on a field study using polysomnography, and therefore, the result was fundamentally different from the other field studies. EEG awakening was defined using polysomnography, which was expected to occur spontaneously 8760 times per year. The effects of the elapsed time after falling asleep, sleep stages and REM sleep were accounted for in the relationship.

The three dose-response relationships are expressed as follows:

(8) $$\begin{array}{cc} P_{\mathrm{single},P}= & 1.909\times 10^{-6}\;{\text{SEL}}^{2}-5.64\times 10^{-3} \end{array}$$

(9) $$\begin{array}{cc} P_{\mathrm{single},A}= & \frac{1}{1+\exp\left\{-\left(-6.8884+0.04444\text{SEL}\right)\right\}} \end{array}$$

(10) $$\begin{array}{cc} P_{\mathrm{single},B}= & 1.89\times 10^{-5}L_{\max}^{2}+4.01\times 10^{-4}L_{\max}-3.3243\times 10^{-2} \end{array}$$

where Equations (8)–(10) are confined to SEL >54 (dB), SEL >50 (dB) and $L_{\max}>32$ (dB), respectively.

In these relationships, a commercial aircraft was assumed to be the sound source. Passchier-Vermeer reported a relationship of noise indices as follows:

(11) $${\text{SEL}}_{10}=16.40+0.877L_{\max}$$

where SEL${}_{10}$ is the equivalent sound level of a noise event normalized to 1 s and assessed over the time the sound level of the noise event was larger than $L_{\max}-$ 10 (dB). The values of SEL and SEL${}_{10}$ are similar, and therefore, Equations (8) and (9) were converted to:

(12) $$\begin{array}{cc} P_{\mathrm{single},P}= & 1.468\times 10^{-6}L_{\max}^{2}+5.491\times 10^{-5}L_{\max}-5.13\times 10^{-3} \end{array}$$

(13) $$\begin{array}{cc} P_{\mathrm{single},A}= & \frac{1}{1+\exp\left\{-\left(-6.1596+0.03897L_{\max}\right)\right\}} \end{array}$$

where Equations (8) and (9) are confined to $L_{\max}>43$ (dB) and $L_{\max}>38$ (dB).

**Figure 2:**
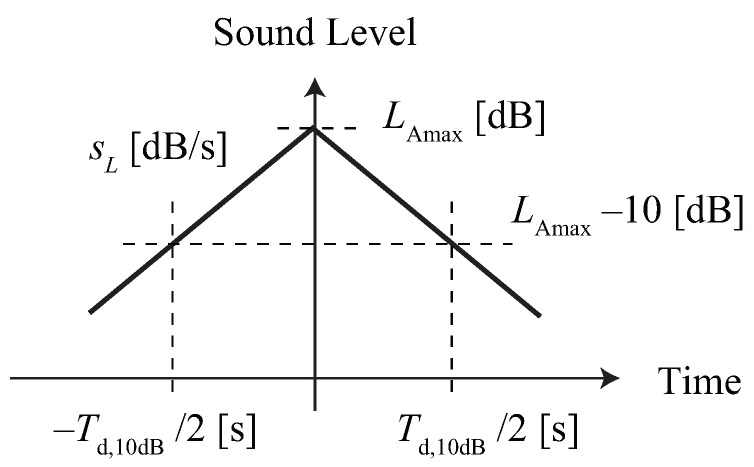
Assumed fluctuation of a single noise event, where sound level linearly increases and decreases. The slope of a single noise event ($s_{L}$) and the 10 dB-duration of a single noise event ($T_{d,10\mathrm{dB}}$) correspond to Equation (11).

The following power function was assumed as $p_{\mathrm{unit}}\left(L\left(t\right)\right)$;

(14) $$p_{\mathrm{unit}}\left(L\left(t\right)\right)=a{\left(L\left(t\right)-b\right)}^{c}$$

where, the symbols *a* and *c* are constants and *b* is the threshold level of awakening risk. A least-square method was used to determine the set of constants using Equations (8), (9) or Equation (10) and Equation (7), where linearly increasing and decreasing single noise events were assumed. Figure 2 shows the assumed fluctuation of the sound level. The integral calculation of $p_{\mathrm{unit}}$ in Equation (7) was carried out using $L_{\mathrm{Amax}}-30$ (dB) to account for the effect of the low sound level. The $s_{L}$ and the $T_{d,10\mathrm{dB}}$ were determined as follows:

(15) $$\begin{array}{cc} s_{L} & =10^{0.0123L_{\max}-0.747} \end{array}$$

(16) $$\begin{array}{cc} T_{d,10\mathrm{dB}} & =10^{-0.0123L_{\max}+2.048} \end{array}$$

which correspond to Equation (11).

The constant *b* was set to the threshold levels used in Equations (12), (13) or Equation (10), respectively.

### 2.2. Redefinition of the $N_{\mathrm{awake},\mathrm{year}}$ Index

The $p_{\mathrm{unit}}$ was defined as the probability of awakening due to a single noise event, which was equivalent to the probability calculated using the existing relationship. However, since chronic adverse health effects are not induced by a single noise event and may be caused by long-term noise exposure, the long-term night-time noise index, $N_{\mathrm{awake},\mathrm{year}}$, is addressed in this section.

While the $L_{\mathrm{night}}$ over- or under-estimates the number of awakenings in response to noise events during night-time, the $N_{\mathrm{awake},\mathrm{year}}$ index considers the expected number of awakenings (or probability of awakening) per year, which would be appropriate since awakenings during sleep might cause adverse health effects. This index has already been defined as a summation of the awakening probability due to a single noise event calculated using equations, such as Equations (8), (9) or Equation (10). However, the calculation can only be performed under limited circumstances because the equation for the probability of awakening is confined to a specific sound source.

The total number of awakenings per year is obtainable when the calculation is based on the awakening potential introduced in the previous section. The low volume of night-time traffic enables the associated noise level to be measured separately as a single event. Therefore, each awakening potential due to a single noise event can be summed up to estimate the total awakening potential during the night. Furthermore, the total awakening potential corresponded to the total number of awakenings per year and were expressed as follows:

(17) $$\begin{array}{cc} N_{\mathrm{awake},\mathrm{year}} & =\sum_{\mathrm{night},\mathrm{year}}P_{\mathrm{single}} \end{array}$$

(18) $$\begin{array}{cc} & =\int_{\mathrm{night},\mathrm{year}}p_{\mathrm{unit}}\left(L\left(t\right)\right)\;dt \end{array}$$

(19) $$\begin{array}{cc} & =\sum_{\mathrm{night},\mathrm{year}}g\left(\int_{\mathrm{event}}f\left(L\left(t\right)\right)\;dt\right) \end{array}$$

A significant point worth emphasizing is that we included neurophysiological determinants into the redefined $N_{\mathrm{awake},\mathrm{year}}$ in Equation (18). The neurophysiological meaning of this definition is shown in Equation (19). Sound level input into the brainstem (L(t)) was converted to electrical external stimuli (function *f*) and integrated into the brainstem, though there is an assumption where the integration system of the brainstem could be approximated to a simple integration system, since night-time noise is relatively short. Integrated electrical external stimuli determine the probability of awakening in response to a single noise event (function *g*), and $N_{\mathrm{awake},\mathrm{year}}$ is the sum of the probability. Awakening potential (function $p_{\mathrm{unit}}$) is a composite function of *f* and *g* as mentioned in the previous section. This redefinition also implies that $N_{\mathrm{awake},\mathrm{year}}$ can be defined in various single noise events despite specific noise events, such as the noise of a commercial aircraft.

Simulated calculations based on the existing dose-response relationship between SEL and the awakening probability, depicted in Equation (8), were performed to examine the validity of the developed noise index. The Passchier-Vermeer and ANSI relationships shown in Equations (8) and (9) were used in this calculation. Some assumptions were made to perform the calculation: sound levels that increase and decrease linearly during a noise event, 1–50 noise events during a night and the difference in sound levels between outdoors and indoors of 15 (dB) [31].

### 2.3. Application: Community Noise in a Suburb

Two examples of the application of the redefined $N_{\mathrm{awake},\mathrm{year}}$ index are presented based on the sound level measurements of traffic noise. The Passchier-Vermeer, ANSI and Basner relationships shown in Equations (8)–(10) were used in this calculation.

**Figure 3:**
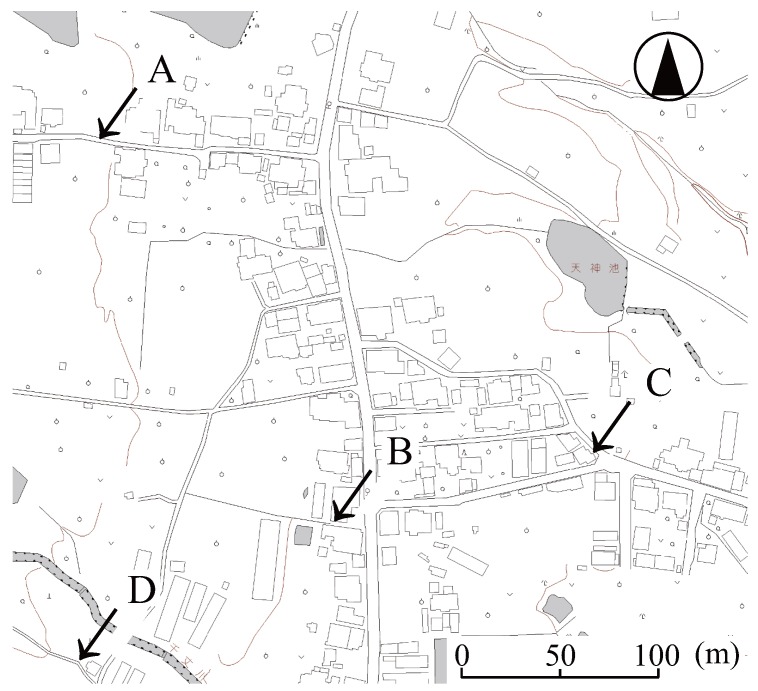
Measurement points in a Kyoto city suburb (Ooenishinaga-chou, Nishikyou-ku, Kyoto city, Japan). Measured Point A was located alongside a quiet road and opened to an urban road where a number of automobiles passed through; B was located alongside the urban road; C was located near a Y-shaped intersection where a few automobiles passed through and opened to the urban road; and D was located in a residential district and not opened to any major roads.

Community noise measurements were carried out in a suburb of Kyoto City for 24 h [32], and noise indices including the redefined $N_{\mathrm{awake},\mathrm{year}}$ index were calculated. The four measured points are shown in Figure 3. At these points, the major sound sources were several automobiles passing through an urban road that lies at the center of the figure. The difference between indoor and outdoor noise levels was set at 15 (dB).

### 2.4. Application: Effect of a Noise Barrier along a Motorway

**Figure 4:**
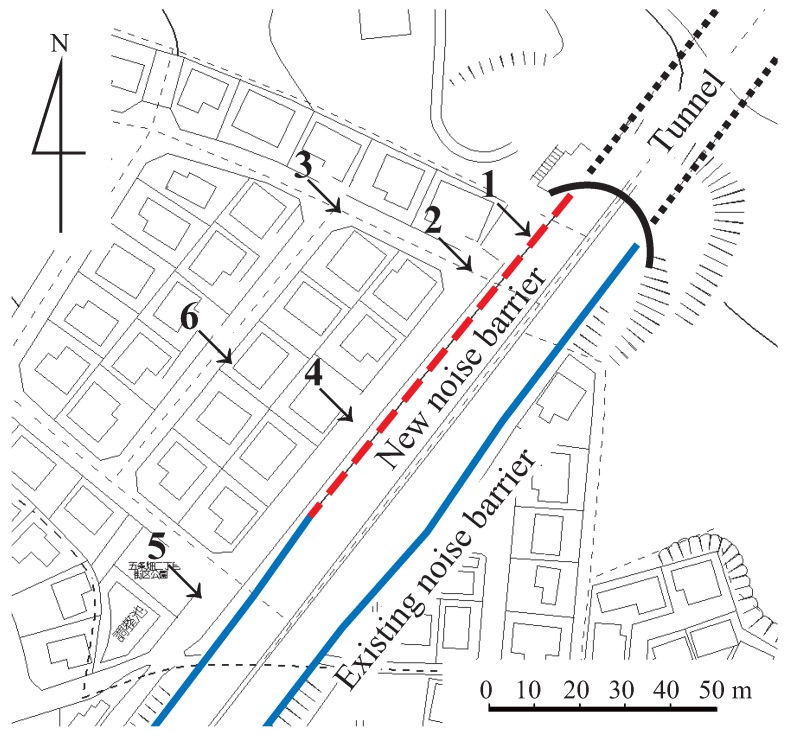
Measurement points alongside a trunk road of the “Daini Hanna Toll Road” in Nara city (Naka-machi, Nara City, Japan). Sound levels were measured before and after setting up a noise barrier (broken line).

An additional noise measurement was performed along a trunk road of the “Daini Hanna Toll Road” in Nara City (see Figure 4). Noise measurements were conducted at six points along the motorway before and after a new barrier was set up to reduce the traffic noise there. Detailed information of the measurement is shown below:

- Instruments: high-precision sound level meter, NL-31, RION (A-weighted, fast, 0.1-s interval sampling) and digital recorder, R-09, Roland , with a binaural microphone, BME-200, ADPHOX (pulse-code modulation (PCM) recording).
- Date: 1:00–4:00 a.m. 28 November 2007 (before), 1:00–4:00 a.m. 29 February 2008 (after).
- Procedure: Successive 20-min noise measurement and audio recording were performed at each point, respectively. We determined a dominant sound source every moment [32], and night-time noise indices were calculated with the available data where the dominant sound source was road traffic noise.

The $L_{\mathrm{night}}$ and redefined $N_{\mathrm{awake},\mathrm{year}}$ were calculated at these 6 measurement points, where the difference between indoor and outdoor noise level was set at 15 (dB).

## 3. Results

### 3.1. Overview of $p_{\mathrm{unit}}$

The formula for determining the $p_{\mathrm{unit}}\left(L\left(t\right)\right)$ (s${}^{-1}$) as the function of indoor sound level L(t) was obtained from each existing study, as follows:

(20) $$\begin{array}{ccc} p_{\mathrm{unit},P} & =4.545\times 10^{-6}{\left(L\left(t\right)-43\right)}^{1.300} & \text{for}\;43\;\mathrm{dB}\leq L(t) \end{array}$$

(21) $$\begin{array}{ccc} p_{\mathrm{unit},A} & =6.972\times 10^{-6}{\left(L\left(t\right)-39\right)}^{1.610} & \text{for}\;39\;\mathrm{dB}\leq L(t) \end{array}$$

(22) $$\begin{array}{ccc} p_{\mathrm{unit},B} & =5.617\times 10^{-6}{\left(L\left(t\right)-32\right)}^{1.831} & \text{for}\;32\;\mathrm{dB}\leq L(t) \end{array}$$

**Figure 5:**
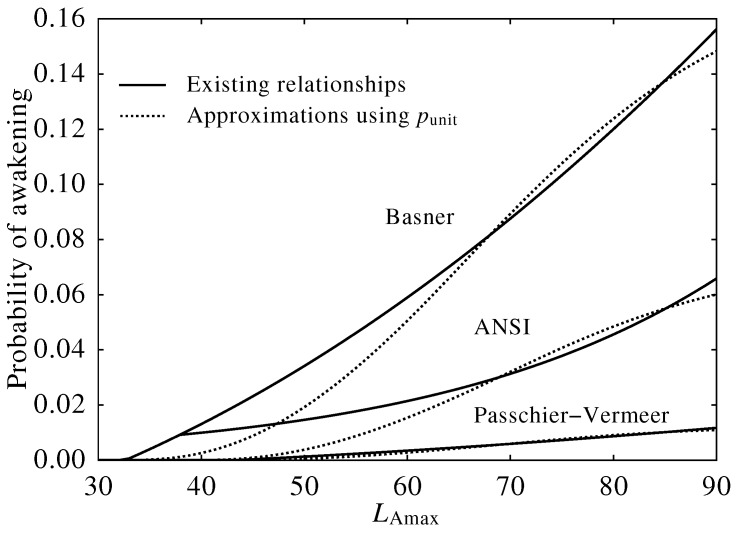
Dose-response relationships between maximum sound level per event ($L_{\max}$) and the probability of awakening. Each solid line was calculated from an existing dose-response relationship expressed by Equations (12), (13) or Equation (10), and each dotted line was calculated from the integration of awakening potential using Equation (7) with Equations (20), (21) or Equation (22). ANSI, American National Standards Institute.

Figure 5 shows the dose-response relationship between $L_{\max}$ and the probability of awakening calculated by the existing relationships depicted in Equations (12), (13) or Equation (10) against the approximations using the integral calculation of the $p_{\mathrm{unit}}\left(L\left(t\right)\right)$ depicted in Equation (7) with Equations (20), (21) or Equation (22). It should be noted that the ANSI relationship between the sound level and the probability of awakening depicted in Equation (13) is confined to $L_{\max}>39$ (dB), which is equivalent to SEL>50 (dB). Therefore, the relationship is discontinuous at the value of the threshold, while the developed function does not include this function.

For each set of existing and approximated relationships, the dose-response curves agree substantially, which means that the approximated relationships could be used as a substitute for the existing one. However, there are a few qualitative differences between each existing and approximated curve, which can be described as follows.

1. Each approximated curve has an inflexion point at the high sound level since the duration of a single noise event calculated using Equation (16) is short when the maximum sound level is high.
2. The probability of awakening calculated using Equation (13) is discontinuous at the threshold of 39 (dB), since a logistic function was selected to understand the probability of awakening in the ANSI method.
3. The probability of awakening calculated using Equation (10) rises steeply to the threshold, since this curve was derived by subtracting a constant value from a logistic function.

These differences would arise partly because of the assumptions made in this study and partly because of the analytical methods used in previous studies.

### 3.2. $N_{\mathrm{awake},\mathrm{year}}$ Index

Three equations of the $p_{\mathrm{unit}}$, Equations (20)–(22), cover a wide range of single noise events, because they are based on the neurophysiological evidence garnered from sleep studies. These equations were first derived from Equations (8)–(10), which means that the number of awakenings per year could be estimated by redefining the $N_{\mathrm{awake},\mathrm{year}}$ expressed as Equation (18).

**Figure 6:**
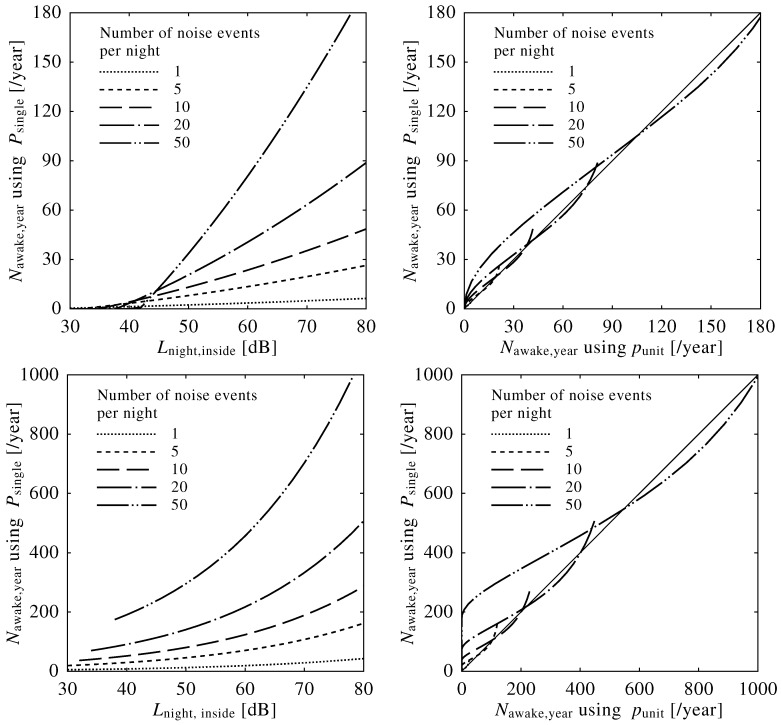
Night equivalent level ($L_{\mathrm{night}}$) *versus* number of awakenings per year from simulated calculations based on $P_{\mathrm{single}}$ (Equation (17)) with the Passchier-Vermeer equation (Equation (8), upper left panel) and the ANSI equation (Equation (9), lower left panel). Comparison of the number of awakenings redefined based on $p_{\mathrm{unit}}$ (Equation (18)) with approximation of the Passchier-Vermeer equation (Equation (20)), upper right panel) and the ANSI equation (Equation (21), lower right panel). Calculations were performed with various numbers of noise events from 1–50 times per night.

Figure 6 shows the results of the simulated calculations based on the $L_{\mathrm{night}}$, as well as the existing and defined $N_{\mathrm{awake},\mathrm{night}}$ index.

The left panels of Figure 6 show the relationship between $N_{\mathrm{awake},\mathrm{night}}$ based on $P_{\mathrm{single}}$ depicted in Equation (17)) and $L_{\mathrm{night}}$. Since the probability of awakening is calculated as zero if the SEL is below 50 (dB) in the ANSI equation, the number of awakenings per year based on Equation (17) is discontinuous when $L_{\mathrm{night}}$ is low and the number of sound events is high. There are remarkable differences between the curves, where the number of awakenings is very high when the number of noise events is high, even if the $L_{\mathrm{night}}$ was calculated to be the same value.

The right panels in Figure 6 show the relationship between the $N_{\mathrm{awake},\mathrm{night}}$ based on $P_{\mathrm{single}}$ and $p_{\mathrm{unit}}$ depicted in Equations (17) and (18), respectively, where the former is the existing definition and the latter is the redefinition. The redefined $N_{\mathrm{awake},\mathrm{year}}$ is larger than the existing definition of $N_{\mathrm{awake},\mathrm{year}}$ based on the ANSI equation, because of their mathematical difference of continuity at the value of threshold as described above. However, each pair of the estimations agrees strongly, even if the number of noise events varies widely.

### 3.3. Application: Community Noise in a Suburb

Night-time noise indices (22:00–6:00) at measurement points shown in Figure 3 are listed in Table 1.

It should be noted that the $N_{\mathrm{awake},\mathrm{year}}$ index based on the Passchier-Vermeer and the ANSI relationships was established with the probability of behavioral awakenings while the $N_{\mathrm{awake},\mathrm{year}}$ index based on the Basner relationship was associated with the probability of EEG awakening. This means that the latter value is fundamentally different from the others. Basner [8] reported that spontaneous EEG awakenings can be expected up to 8760 times per year, and the threshold value is very low.

The difference between Points B and C is only 4 (dB), but the awakening risk at Point B was 4–8-times higher than that at Point C was when the calculation of awakening was based on the Passchier-Vermeer or ANSI relationship, and the risk of sleep disturbance is considered significantly high. The $N_{\mathrm{awake},\mathrm{year}}$ appeared to have a distinct advantage for evaluating night-time noise, as well as sleep disturbance.

In contrast, the risk order is reversed and is almost the same when the calculation is based on the Basner relationship. This means that the risk of sleep disturbance varies significantly depending on whether it is caused by behavioral or EEG awakening. Furthermore, epidemiological studies may reveal appropriate end-points and the $N_{\mathrm{awake},\mathrm{year}}$ index as mentioned in the previous section.

In addition, at all pf the measurement points, the sound levels evaluated using the $L_{\mathrm{night}}$ and the $L_{\mathrm{Amax}}$ were higher than the WHO guideline values [17] and the EU night-time noise guideline (40 (dB)) [4]. However, the $N_{\mathrm{awake},\mathrm{year}}$ index at Point D showed a low risk of sleep disturbance.

### 3.4. Application: Effect of a Noise Barrier along a Motorway

**Figure 7:**
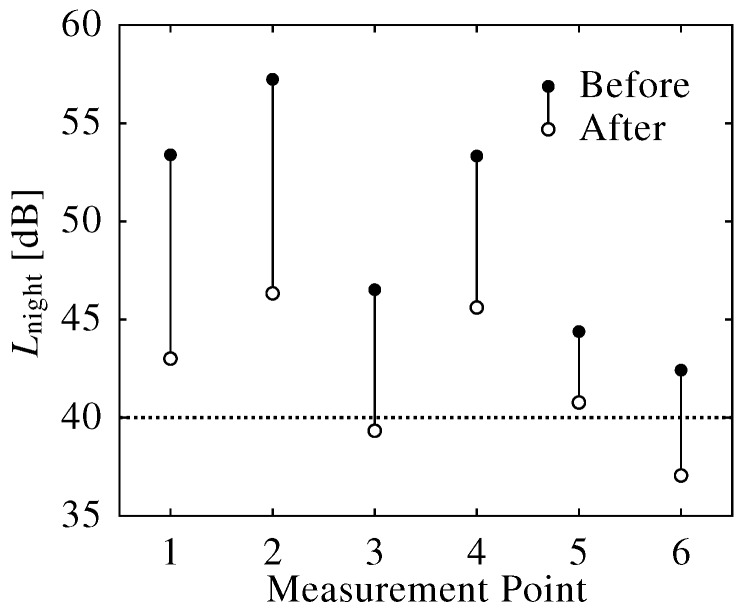
Decrease in night equivalent level ($L_{\mathrm{night}}$) by the noise barrier.

Figure 7 shows the reduction of traffic noise evaluated using $L_{\mathrm{night}}$ (dB) (22:00–6:00). Although the $L_{\mathrm{night}}$ decreased by 5–10 (dB) after the noise barrier was set up, we were unable to evaluate the mitigation of sleep disturbance from these measurements alone.

**Figure 8:**
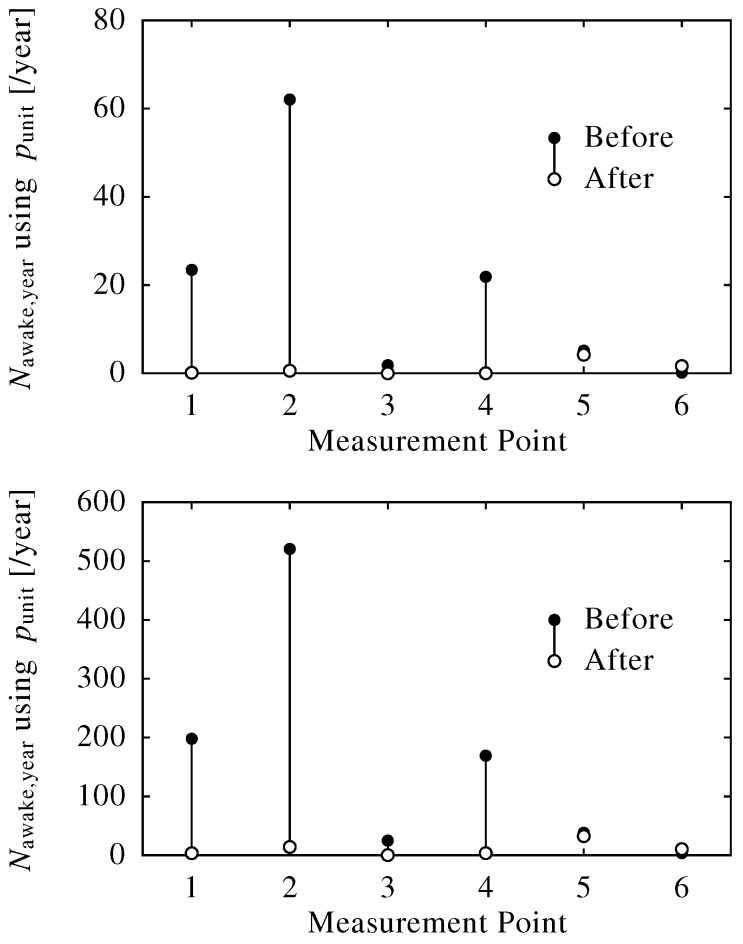

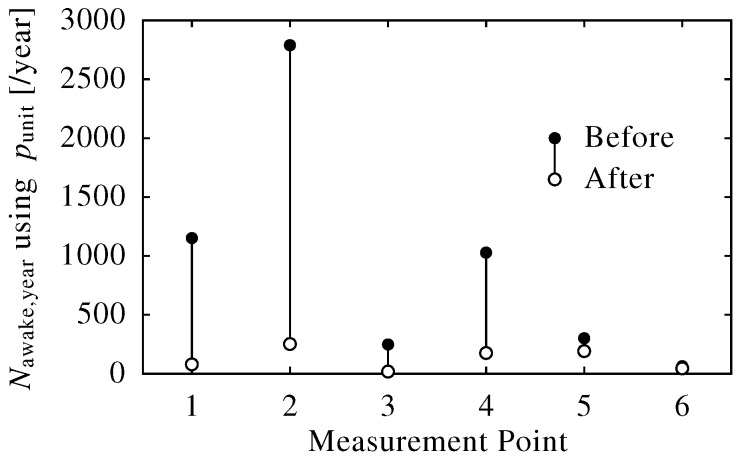
Decrease in $N_{\mathrm{awake},\mathrm{year}}$ by the noise barrier. $N_{\mathrm{awake},\mathrm{year}}$ was calculated based on awakening potential unit ($p_{\mathrm{unit}}$) with the approximation of the Passchier-Vermeer, ANSI and Basner equation (Equations (20)–(22) in the upper, middle and lower panels), respectively.

Meanwhile, Figure 8 shows the reduction of the number of awakenings using $N_{\mathrm{awake},\mathrm{year}}$ (year${}^{-1}$) (22:00–6:00). The $N_{\mathrm{awake},\mathrm{year}}$ were decreased and were almost zero at all measurement points after the barriers were set up, which means the awakening risk along this road decreased greatly and the effect of noise barriers was beneficial, even though the $L_{\mathrm{night}}$ at some measurement points after the barriers were set up was higher than the guideline value of 40 dB.

## 4. Discussion

The aim of this study is to introduce a neurophysiologically-validated noise index for evaluating awakenings in response to noise events, although currently-used indices, such as SEL, $L_{\mathrm{Amax}}$ and $L_{\mathrm{night}}$, were introduced mainly because of the statistically high correlation with sleep effects.

Simulated calculations [22] based on the mathematical modeling of the brainstem [25,26] revealed that the brainstem integrates awakening potential, but not the sound energy of external stimuli. In addition, the time constant integrating the potential was revealed to be approximately 10–100 s when a first-order lag system was assumed. This evidence facilitated the introduction of a mathematical $p_{\mathrm{unit}}$, as well as the redefinition of the $N_{\mathrm{awake},\mathrm{year}}$ index based on an integral calculation of the awakening potential unit depicted in Equation (18).

The $p_{\mathrm{unit}}$, as a function of the sound level depicted in Equations (20), (21) or Equation (22), was derived using the existing dose-response relationship between the established SEL or $L_{\mathrm{Amax}}$ index and the probability of awakening due to a single noise event depicted in Equations (12), (13) or Equation (10). Some assumptions were made in this study, including that the sound level linearly increased and decreased during the noise event, and its duration was determined by the maximum level. Although there are some small gaps between the existing and the approximation curves, these likely appeared partly because of the assumptions made in this study and partly because of the analytical methods used in the previous study. The approximated curves could be modified to fit the existing ones by selecting another function or threshold; however, it is more appropriate to modify the $p_{\mathrm{unit}}$ based on the existing or new studies where the sound level fluctuation is available, since some assumptions were made in this study to derive the approximations.

In addition, a variable that provides an explanation for the distribution of individual noise events over one night (*i.e.*, elapsed time of falling asleep) should be accounted for in future studies, as ANSI [18] and Basner *et al.* [8]. Neurophysiologically, the existence of the circadian rhythm was accepted [23], and the Phillips-Robinson model [25] also includes a term of the circadian rhythm that fluctuates as a sinusoidal curve, which considerably affects the thresholds of awakening.

The redefined $N_{\mathrm{awake},\mathrm{year}}$ index provided the expected total number of awakenings per year. As shown in Figure 6, simulated calculations were carried out to compare the validity of the $N_{\mathrm{awake},\mathrm{year}}$ and the $L_{\mathrm{night}}$, where a wide variety of the number of noise events during a night was assumed. The $N_{\mathrm{awake},\mathrm{year}}$ index strongly agreed with the number of awakenings estimated from the simulated calculations, while the $L_{\mathrm{night}}$ induced remarkable variation in the relationship. The redefined $N_{\mathrm{awake},\mathrm{year}}$ is based on neurophysiological sleep parameters and covers a wide-range of cases associated with various types of noise.

Examples of the application of the redefined $N_{\mathrm{awake},\mathrm{year}}$ were presented using the measurements of community noise. The $N_{\mathrm{awake},\mathrm{year}}$ appeared to have distinct advantages in evaluating night-time noise, as well as sleep disturbance compared to the other indices. The application of the $N_{\mathrm{awake},\mathrm{year}}$ in epidemiological studies on adverse health effects would evaluate more efficiently than the $L_{\mathrm{night}}$ index would. In addition, countermeasures to mitigate deleterious health effects could be applied effectively.

The total number of awakenings was also adopted by the ANSI [18] for evaluating cumulative risk of noise-induced sleep disturbance. It has the advantage of showing the awakening responses of residents directly, as opposed to the $L_{\mathrm{night}}$ index, which shows just an average sound level in dB. Moreover, our epidemiological study [33] showed that the risk of hypertension around two different airfields could be consistently evaluated using the $N_{\mathrm{awake},\mathrm{year}}$ defined by the ANSI, but not the $L_{\mathrm{night}}$ or day-evening-night equivalent level ($L_{\mathrm{den}}$), where the number, maximum sound level and duration of noise events were different between the two airfields.

We propose that epidemiological studies using the $N_{\mathrm{awake},\mathrm{year}}$ index may reveal more reasonable dose-response relationships between subjective sleep disturbance and adverse health effects than the existing index of $L_{\mathrm{night}}$. In addition, since the $N_{\mathrm{awake}}$ was defined neurophysiologically in this study, it may provide a consistent explanation for risks of aircraft, road traffic and railway noise where characteristics of noise exposure are quite different though the risks of road traffic and railway noise were not examined in this study. Further studies are required to elucidate and appropriately modify the functionality of the $p_{\mathrm{unit}}$ and the $L_{\mathrm{night}}$ in evaluating night-time noise-induced sleep disturbances. In addition, the effects of the distribution and sound sources of noise events should be examined using the $N_{\mathrm{awake},\mathrm{year}}$.

## 5. Conclusions

This study proposes the total number of awakenings per year ($N_{\mathrm{awake},\mathrm{year}}$) as a night-time noise index based on the neurophysiological evidences elucidating the awakening process in the brainstem. The $N_{\mathrm{awake},\mathrm{year}}$ is calculated by integrating the awakening potential ($p_{\mathrm{unit}}$) introduced neurophysiologically and derived using the existing dose-response relationship between sound levels with the probability of awakening, which has distinct advantages in evaluating night-time noise, as well as sleep disturbance compared to the other indices.

## Figures and Tables

**Table 1:** Night equivalent noise level ($L_{\mathrm{night}}$), maximum sound level ($L_{\mathrm{Amax}}$) and $N_{\mathrm{awake},\mathrm{year}}$ night-time noise indices measured at the specific location of a Kyoto city suburb illustrated in Figure 3. The $N_{\mathrm{awake},\mathrm{year}}$ index was calculated using an integral calculation of the $p_{\mathrm{unit}}$ derived from the Passchier-Vermeer, ANSI and Basner equations. The difference between the indoor and outdoor sound level was set at 15 (dB).

		Measured Point			
Index		A	B	C	D
$L_{\mathrm{night},\mathrm{outside}}$ (dB)		49.8	55.7	51.7	46.0
$L_{\mathrm{Amax},\mathrm{outside}}$ (dB)		86.6	84.1	80.0	70.7
$N_{\mathrm{awake},\mathrm{year}}$ (year${}^{-1}$)	using the Passchier-Vermeer equation	3.7	35.3	3.9	0.2
	using the ANSI equation	21.4	205.7	49.1	1.2
	using the Basner equation	76.8	733.6	829.1	44.0
